# Influence of Surgical Expertise on Repair of Acute Type a Aortic Dissection in a Standardized Operative Setting

**DOI:** 10.3390/jcm14061819

**Published:** 2025-03-08

**Authors:** Daniela Piani, Sandro Sponga, Andrea Lechiancole, Gregorio Gliozzi, Stefano Copetti, Arianna Semeraro, Elisabetta Auci, Uberto Bortolotti, Ugolino Livi, Igor Vendramin

**Affiliations:** 1 Cardiothoracic Department, Azienda Sanitaria Universitaria Friuli Centrale, 33100 Udine, Italyigor.vendramin@asufc.sanita.fvg.it (I.V.); 2 Department of Medicine, University of Udine, 33100 Udine, Italy; 3 Department of Anesthesiology, Azienda Sanitaria Universitaria Friuli Centrale, 33100 Udine, Italy

**Keywords:** acute aortic dissection, ascending aorta, aortic arch, early and late results

## Abstract

**Background/Objectives:** The influence of surgeon expertise on patients’ outcomes after repair of acute type A aortic dissection (ATAAD) is not well established. The aim of this paper is to report the results of ATAAD repair performed by expert (ES) and not expert aortic surgeons (NES) in our center. **Methods**: We have retrospectively divided 199 patients into two groups according to the first surgeon experience (ES = 138 patients and NES = 61 patients), all being members of the aortic team. We evaluated and compared early and long-term outcomes. **Results**: Although the two groups were comparable in terms of clinical presentation and intraoperative setting, ES performed more aortic arch repairs (40% vs. 26%, *p* = 0.06) and frozen elephant trunk procedures (15% vs. 3%, *p* = 0.02), albeit with similar intraoperative times (median cardiopulmonary bypass time of 203 min in ES vs. 201 min in NES, respectively, *p* = 0.88). The 30-day mortality was the same in the two groups (8%, *p* = 1), and the postoperative course was similar except for a shorter in-hospital stay in the NES group (10 vs. 17 days, *p* = 0.04). **Conclusions**: In our experience, repair of ATAAD could be achieved with similar results between ES and NES. However, NES performed less technically demanding repairs. With standardization of pre-, intra-, and post-operative management, NES can be expected to increase their technical skills and be progressively involved in more complex ATAAD repairs without adversely affecting surgical results.

## 1. Introduction

Despite the improvements made in recent years in diagnosis and management, acute type A aortic dissection (ATAAD) remains a life-threatening disease [1], with a mortality of 0.5%/h and over 23% in the first 48 h in medically treated patients [2]. It has been described that one of every five patients with ATAAD die before hospital arrival, one of three within 24 h, and over half of all patients within 30 days [3].

Patients’ outcomes following repair of ATAAD depend on many factors, such as age, clinical instability, presence of cardiac tamponade with shock, and organ malperfusion, particularly with cerebral damage [4,5,6]. Surgical experience also plays an important role, since selection of the operative strategy in ATAAD repair is crucial for patient outcome. In fact, it has been shown that mortality for ATAAD is lower when repair is performed in high-volume rather than in low-volume centers by low-volume surgeons [7,8].

Recent guidelines define a high-volume aortic center as one that performs ≥7 aortic root, ascending aorta, or transverse aortic arch dissection repairs per year [9]. However, since this definition may be somewhat arbitrary, as that concerning expert (ES) and non-expert surgeons (NES), this issue is still a matter of debate.

In the present article, we aimed to verify whether the results of ATAAD repair were influenced by the expertise of surgeons of our aortic team.

## 2. Materials and Methods

### 2.1. Study Population

The present study, which considers a time span between January 2010 and December 2021, enrolled a total of 199 consecutive patients who underwent surgery for ATAAD at our Center. We have divided the patients into two groups: those operated on by ES (*n* = 138) and those operated on by NES (*n* = 61).

Preoperative, demographic, intraoperative, and postoperative data were collected.

Acute kidney injury was defined by the increase in serum creatinine to 1.5 times from baseline, according to KDIGO [10].

### 2.2. Center and Surgeon Characteristics

Our center performs an average of 20 ATAAD repairs per year. The aortic team, founded in 2010, manages all patients presenting with acute or chronic aortic pathologies; at the time of the present review, the aortic team included 8 cardiac surgeons, among whom there were 3 ES and 5 NES, as previously defined according to the consistency of their volume activity [9]: ES performed at least 100 cardiac operations per year and a minimum of 7 ATAAD repairs, while NES were those in their first 10 years of practice or with limited expertise on aortic surgery, both in acute settings (<7 ATAAD repairs per year) and in elective surgeries for arch diseases. The surgical procedure was performed either by an ES or an NES and an assisting surgeon depending on the on-call shift, regardless of the clinical urgency or the dissection’s anatomical characteristics.

Since 2016, a systematic approach has been adopted in the treatment of ATAAD patients by creating a regional network and multidisciplinary teamwork with the goal of increasing early referral, simplifying the communication between hub centers (HC) and our spoke center (SC), and providing emergency radiological teleconsultation, thus minimizing the time between presentation and treatment [11].

### 2.3. Patient Evaluation and Management

With clinical suspicion of ATAAD, definitive diagnosis is confirmed by transthoracic 2D-echo and/or angio-computed tomography (CT). Patients with a diagnosis of ATAAD in an SC are immediately referred to our HC, where, in the meantime, the aortic team decides the most suitable therapeutic strategy. In cases of uncertain diagnosis, imaging results are sent from the SC to the HC for consultation with the HC radiologists. Otherwise, if there is no surgical indication, the patients remain in the SC, and clinical and radiological consultation is maintained with the HC.

During transportation from the SC to the HC, continuous anesthesiological assistance is provided with monitoring of vital parameters (central venous pressure, systemic arterial pressure, and urinary output); hemodynamic instability and/or neurological impairment may entail patients’ sedation and intubation. Patients presenting to the HC emergency department are managed according to the same protocol before being transferred to the HC operating room.

### 2.4. Surgical Technique

Since 2010, surgical techniques have been standardized as previously described [12,13]. The preferred arterial site for cannulation is the right axillary artery, which has progressively replaced the femoral artery for the institution of cardiopulmonary bypass (CPB). When the entrance tear is identified in the ascending aorta, and the aortic arch is not dilated, only the ascending aorta and hemiarch are replaced with a supra-commissural graft. When, instead, the entry tear is located in the arch or the arch is significantly dilated even in the absence of tears, repair is extended to the aortic arch. Arch replacement is performed using a tri- or quadrifurcated graft with individual reattachment of the brachiocephalic vessels with or without the classic or frozen elephant trunk technique (FET).

Cerebral protection is obtained through the right axillary artery and antegrade selective perfusion of the left carotid and subclavian arteries under moderate systemic hypothermia (median lower temperature of 26 °C); when femoral artery cannulation is used for CPB, the arch vessels are selectively perfused.

### 2.5. Patient Follow-Up

Hospital survivors are followed by a dedicated team according to an institutional protocol that includes clinical and echocardiographic re-evaluation after 1 and 6 months and then yearly when also control CT scans are obtained. Afterwards, the patients undergo CT scans in the 2nd and 3rd postoperative years and then according to their clinical status. Further clinical information is gathered by phone interviews, medical records, and post-mortem reports to assess the incidence and type of major clinical and aortic-related complications.

### 2.6. Statistical Analysis

Continuous variables were tested for normality with the Kolmogorov–Smirnov test and expressed as median and interquartile range; comparison between groups was performed with the Mann–Whitney U test. Categorical variables are presented as absolute numbers and percentages, and their comparison was performed by Pearson’s χ^2^ tests or Fisher’s exact tests, as appropriate. Survival analysis was obtained using the Kaplan–Meier method and compared using the log-rank test.

In order to explore factors associated with 30-day mortality, univariable and multivariable logistic regressions were performed, estimating odds ratios with 95% confidence intervals. Multivariable analyses included all variables clinically and statistically relevant, taking into account the number of events and potential collinearities.

Analyses were performed using IBM SPSS Statistics 26.0 (IBM Corp., Armonk, NY, USA).

## 3. Results

### 3.1. Patient Characteristics

Baseline characteristics of ES (*n* = 138) and NES (*n* = 61) patients are reported in Table 1. Overall, there were 90 males (65%) with a median age of 63 years in ES group and 37 males (61%) with a median age of 70 years in NES group (*p* = 0.54 and *p* = 0.09). Clinical presentation was similar between the two groups; of note, the rate of patients with focal neurologic damage was higher (albeit not statistically significant) in the ES group (15% vs. 5%, *p* = 0.06).

### 3.2. Surgical Data

Complex aortic arch surgery was performed mainly by ES: out of the 71 arch replacements, 23 were performed with FET (2 by NES and 21 by ES, *p* = 0.02). Intraoperative times were similar between the two groups: median aortic cross-clamp time was 114 min for ES and 106 min for NES (*p* = 0.64) with median CPB duration of 203 min for ES and 201 min for NES (*p* = 0.88). The median total circulatory arrest time was 41 min in ES and 39 min in NES patients (*p* = 0.64); in both groups the right axillary artery was the preferred site for the conduction of CPB.

### 3.3. Perioperative Outcomes

Comparing ES and NES, 30-day mortality was 8% for both groups; most deaths occurred in patients with neurological deficit and cardiogenic shock. The incidence of surgical chest re-exploration for bleeding is slightly higher, but not statistically relevant, in the ES group (8% vs. 18% for NES vs. ES, respectively, *p* = 0.09). New onset of permanent neurological complications was observed in 4% of patients of ES (vs. 0% of NES, *p* = 0.18) (Table 2).

### 3.4. Univariable and Multivariable Analysis

At univariable and multivariable analysis, surgeon expertise did not influence 30-day mortality. Indeed, only preoperative cardiogenic shock (OR 6.4) and length of surgical circulatory arrest (OR 1.01) were found to be independent risk factors for 30-day mortality, while axillary artery cannulation was found to be an independent protective factor (OR 0.18) (Table 3).

### 3.5. Long-Term Outcomes

**Figure 1:**
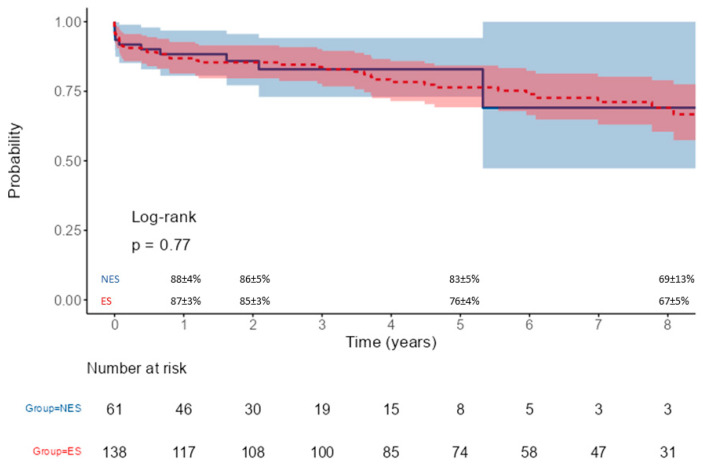
Actuarial survival of patients operated on by non-expert (NES, blue line) and expert (ES, red line) surgeons.

**Figure 2:**
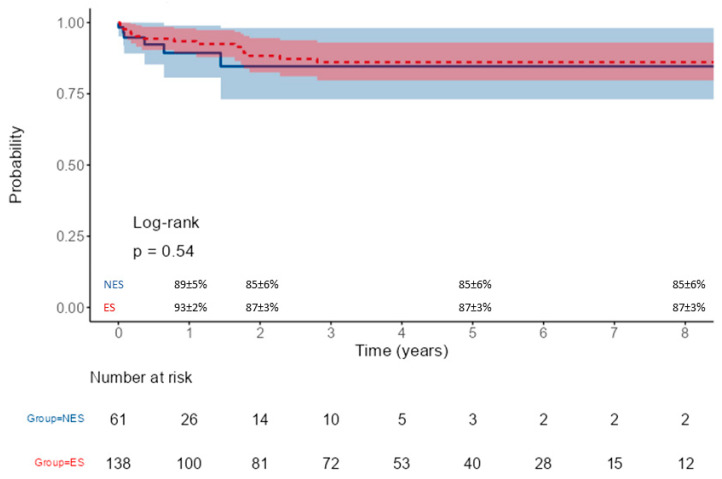
Freedom from reoperation in patients operated on by non-expert (NES, blue line) and expert (ES, red line) surgeons.

Median follow-up was 1.8 years (IQR 1.0–3.8) and 5.3 years (IQR 2.6–7.8) for the NES and ES groups, respectively (*p* < 0.001). Long-term survival was comparable in the two populations, being at 1, 2, and 5 years for the ES group and NES group, 87 ± 3% vs. 88 ± 4%, 85 ± 3% vs. 86 ± 5%, and 76 ± 4% vs. 83 ± 5%, respectively (*p* = 0.77) (Figure 1). During long-term follow-up, 6 patients from the NES group underwent reoperation (three FET, two endovascular procedures, and one proximal reoperation) vs. 15 patients from the ES group (six endovascular procedures, five proximal reoperations, two FET, and two valve operations). Freedom from reoperation is represented in Figure 2. Causes of death were evolution of the aortic dissection in 2 (3%) patients from the NES group and 7 (5%) in the ES group, cardiac failure in 4 (3%) patients form the ES group, and other causes not related to the aortic dissection in 3 patients from the NES group and 15 patients from the ES group.

## 4. Discussion

Although advanced age, preoperative clinical instability, systemic organ malperfusion, and the need for complex repairs have been recognized as significant risk factors adversely influencing surgical results [4,5,6,14,15], the role of surgical expertise in the outcome of ATAAD repair has not been fully evaluated. It has been previously shown that results are improved when repair of ATAAD is performed in high-volume centers and by high-volume surgeons [7,8,16]. Indeed, the Mount Sinai group reported that lower-volume surgeons and centers have approximately double the risk-adjusted mortality than patients undergoing repair by the highest-volume care providers, with institution volume (less than 13 cases a year) and surgeon expertise (less than one ATAAD repair per year) as the strongest predictors of mortality [17]. In others, surgical expertise not only improved early and medium-term outcomes but also allowed higher rates of valve-sparing aortic repairs in ATAAD [18,19]. It has been suggested that outcomes in aortic surgery could be improved in the United States if acute care and surgical treatment of most patients with ATAAD were regionalized and restricted to institutions with high-volume multidisciplinary aortic surgery programs [20]. On the other hand, in the United Kingdom, it has been demonstrated that there is little relationship between volume and outcome at a hospital level, but higher individual surgeon volumes were associated with lower in-hospital mortality [8].

When ATAAD repair is performed by less experienced surgeons, some factors could influence results, such as the possibility of being faced with more stable patients in high-volume centers while having more sick patients in low-volume institutions [21]. Furthermore, it is unclear whether hospital volume can neutralize the surgeon volume or experience, acting therefore as a confounding factor [8]. Large-volume institutions more frequently have a dedicated multidisciplinary aortic team that manages ATAAD patients according to established protocols, which is an important contributing factor to lower mortality in ATAAD repair [22]. Indeed, centralization of ATAAD cases in a high-volume center had a positive impact on lowering mortality despite an insignificant increase in the number of patients treated, as did patient transfer from low- to high-volume centers [23,24,25].

Despite surgical and medical improvements in the treatment of ATAAD, perioperative mortality remains high, being reported to be around 17% in a recent multicenter European registry [13]. In the present experience, we observed a significant reduction in hospital mortality to 8% in the entire series, limiting our analysis to patients operated on during the last decade when we adopted a systematic approach in the treatment of ATAAD by creating a multidisciplinary aortic team. In the same period and with the aim to increase the number of patients receiving an early diagnosis and timely management, we also created a regional network establishing a 24 h connection between our HC and referring SC allowing inter-institutional patient evaluation, radiological teleconsultation, and immediate referral upon diagnosis confirmation. We consider all the aforementioned changes in our center policy and organization as key factors contributing to improving the surgical outcomes of ATAAD patients.

By analyzing the influence of surgical expertise on mortality in the same setting, we have found that, faced with a similar preoperative clinical profile of patients, ES performed more arch replacements with a mean duration of CPB and aortic cross-clamp comparable between the two groups. These results could be related to a difference in the surgeon’s decision-making process since they extended more frequently ATAAD repair to the aortic arch, being more confident with a more complex surgical procedure compared to NES. Considering the postoperative course, our analysis showed that NES patients had a lower incidence (albeit not significant) of permanent neurological damage and a lower hospitalization length with a similar incidence of acute kidney injury. There was a tendency of a higher surgical re-exploration rate for postoperative bleeding in the ES group; this could be related to the higher percentage of redo procedures and aortic arch replacements performed by ES. The 30-day mortality rate of the two groups was 8%; when analyzing the causes of death, it emerged that the patients operated on by expert surgeons suffered mainly from ischemic complications (36%), probably related to the severity and extension of the dissection, while in the NES group, the causes of death were mainly multi-organ failure and heart failure. Despite a higher number of arch procedures by ES, no significant difference was found in the incidence of aortic re-operations with a similar survival of >70% at the 5-year follow-up.

At multivariable analysis, preoperative cardiogenic shock and circulatory arrest time were independent risk factors for mortality; axillary artery cannulation was an independent protective factor, while surgical expertise did not influence 30-day mortality.

We believe that our overall results support the possibility that NES may acquire adequate surgical skills, allowing them to progressively shorten their learning curve, which, in our opinion, may also be a consequence of the standardization in the management of ATAAD in our institution from 2010. Furthermore, the present experience indicates that the adoption of a specific organization model for ATAAD patients, which includes a network connecting several SCs with an experienced HC, provides undelayed diagnosis, aggressive medical management, and expeditious surgical repair, which, together with standardization of surgical protocols and strategies, might have contributed not only to improving our surgical results but also to providing a more adequate surgical training of less experienced operators who may be involved progressively in the management of more difficult and higher-risk cases.

## 5. Limitations

The limitations of the present study are certainly related to its retrospective nature. Moreover, the number of patients included is limited by the fact that we have confined our analysis to the last decade of experience when surgical strategies have been standardized in the repair of ATAAD. This has eliminated, in most cases, the potential biases deriving from each surgeon using different techniques. Furthermore, reproducibility of the procedures, coupled with some specific techniques such as the routine use of right axillary artery cannulation, has allowed even NES to obtain satisfactory results. Moreover, the different length of follow-up between ES and NES patients represents a further limitation in the evaluation of late mortality and need for reoperation.

## 6. Conclusions

In our experience, the overall results in patients undergoing ATAAD repair were satisfactory and not significantly influenced by the surgical experience of the aortic team members. It must be obviously underlined that NES operated on less complicated patients and performed less technically demanding repairs, nevertheless still contributing to the low mortality observed. With the institution of an aortic team and the standardization of the preoperative and intraoperative settings, it can be expected that NES may progressively be involved in the care of more complex ATAAD patients, increase their experience, and improve their technical skills, without adversely affecting outcomes even when extended repairs are scheduled.

## Figures and Tables

**Table 1:** Clinical and surgical data.

	NES Group (*n* = 61)	ES Group (*n* = 138)	*p*-Value
**Clinical profile**			
Male sex, *n*. (%)	37 (61)	90 (65)	0.54
Age, years, median (IQR)	70 (58–78)	63 (55–73)	0.09
Creatinine (mg/dL), median (IQR)	1.00 (0.99–1.23)	1.00 (0.98–1.20)	0.92
Previous cardiac surgery, *n*. (%)	0 (0)	10 (7)	0.03
**Risk factors**			
Treated arterial hypertension, *n*. (%)	46 (75)	106/137 (77)	0.76
Smoke, *n*. (%)	15 (25)	44/137 (32)	0.29
Diabetes, *n*. (%)	5 (8)	6/137 (4)	0.32
Chronic kidney disease, *n*. (%)	7 (12)	25/137 (18)	0.23
Median preoperative creatinine, mg/dL (IQR)	1 (0.98–1.23)	1 (0.97–1.20)	0.81
**Presentation**			
Cardiac tamponade, *n*. (%)	2 (3)	9 (7)	0.51
Shock, *n*. (%)	2 (3)	12 (9)	0.23
Hypotension, *n*. (%)	1 (2)	12 (9)	0.07
Syncope, *n*. (%)	8 (13)	25 (18)	0.38
Focal neurologic damage, *n*. (%)	3 (5)	21 (15)	0.06
Paraplegia/paraparesis, *n*. (%)	0	2 (1)	1
Coma, *n*. (%)	0 (0)	4 (3)	0.31
**Surgical procedures**			
**Aortic arch replacement**, *n*. (%)	16 (26)	55 (40)	0.06
- Elephant trunk	5 (8)	29 (21)	0.03
- Frozen elephant trunk	2 (3)	21 (15)	0.02
**Aortic root replacement**, *n*. (%)			
- Modified Bentall	9 (15)	16 (12)	0.51
- Reimplantation	0 (0)	5 (4)	0.33
CPB time, median (IQR)	201 (168–253)	203 (164–240)	0.88
ACC time, median (IQR)	106 (73–159)	114 (85–140)	0.64
Circulatory arrest, median (IQR)	39 (31–54)	41 (32–51)	0.64
**Operative setting**			
Axillary artery cannulation, *n*. (%)	55 (90)	104 (75)	0.01
Femoral artery cannulation, *n*. (%)	3 (5)	30 (22)	
Central cannulation, *n*. (%)	3 (5)	4 (3)	

NES = non-expert surgeons; ES = expert surgeons; CPB = cardiopulmonary bypass; ACC = aortic cross-clamp; IQR = interquartile range.

**Table 2:** Early results.

	NES Group (*n* = 61)	ES Group (*n* = 138)	*p*-Value
30-day mortality, *n*. (%) - Hemorrhagic shock, *n*. (%) - Cardiac, *n*. (%) - Ischemic complications, *n* (%) - MOF, *n*. (%)	5 (8) 1 (20) 2 (40) 0 2 (40)	11 (8) 3 (27) 3 (27) 4 (36) 1 (10)	1 0.97 0.26 0.20 0.41
Chest re-exploration, *n*. (%)	5 (8)	25 (18)	0.09
Creatinine peak, (mg/dL), median (IQR)	1.75 (1.47–3.0)	1.84 (1.40–3.0)	0.80
AKI, *n*. (%)	27 (44)	58 (42)	0.77
Dialysis, *n*. (%)	11 (18)	22 (16)	0.73
Neurologic complications, new onset			
- Coma, *n*. (%)	0 (0)	2 (2)	1
- Permanent neurologic focal deficit, *n*. (%)	0 (0)	6 (4)	0.18
- Paraplegia, *n*. (%)	1 (2)	2 (2)	1
Median ICU stay, days (IQR)	2 (0–5)	4 (2–9)	0.004
Median hospital stay, days (IQR)	10 (0–21)	17 (10–28)	0.04

NES = non-expert surgeons; ES = expert surgeons; AKI = acute kidney injury; ICU = intensive care unit; IQR = interquartile range.

**Table 3:** Univariable and multivariate analysis.

	Univariable		Multivariate	
	OR (95% CI)	*p*-Value	OR (95% CI)	*p*-Value
ES group ES vs. NES group	0.97 (0.32–2.92)	0.96		
Age	1.02 (0.98–1.07)	0.34		
Male gender	2.62 (0.72–9.53)	0.14		
Creatinine	0.27 (0.03–2.29)	0.22		
Previous cardiac surgery	1.28 (1.52–10.81)	0.82		
Tamponade/shock	8.44 (2.60–27.42)	<0.001	6.41 (1.71–24)	0.01
Syncope	0.70 (0.15–3.24)	0.65		
Preop. neurological damage	3.47 (1.16–10.33)	0.03		
CPB time	1.01 (1.00–1.01)	0.004		
ACC time	1.00 (1.00–1.02)	0.02		
Circulatory arrest	1.03 (1.00–1.05)	0.01	1.01 (0.99–1.02)	0.04
Axillary vs. femoral artery use	0.22 (0.08–0.65)	0.01	0.18 (0.05–0.66)	0.01

## Data Availability

The data that support the findings of this study are available from the corresponding author upon reasonable request.

## Abbreviations

The following abbreviations are used in this manuscript:

ATAAD	Acute type A aortic dissection
ES	Expert surgeon
NES	Non-expert surgeon
CT	Computed tomography
SC	Spoke center
HC	Hub center
CPB	Cardiopulmonary bypass
IQR	Interquartile range
OR	Odds ratio
FET	Frozen elephant trunk
